# Temporal dynamics of immune-stromal cell interactions in fracture healing

**DOI:** 10.3389/fimmu.2024.1352819

**Published:** 2024-02-22

**Authors:** Christina A. Capobianco, Kurt D. Hankenson, Alexander J. Knights

**Affiliations:** ^1^ Department of Orthopaedic Surgery, University of Michigan, Ann Arbor, MI, United States; ^2^ Department of Biomedical Engineering, University of Michigan, Ann Arbor, MI, United States

**Keywords:** fracture healing, osteoimmunology, inflammation, bone, crosstalk

## Abstract

Bone fracture repair is a complex, multi-step process that involves communication between immune and stromal cells to coordinate the repair and regeneration of damaged tissue. In the US, 10% of all bone fractures do not heal properly without intervention, resulting in non-union. Complications from non-union fractures are physically and financially debilitating. We now appreciate the important role that immune cells play in tissue repair, and the necessity of the inflammatory response in initiating healing after skeletal trauma. The temporal dynamics of immune and stromal cell populations have been well characterized across the stages of fracture healing. Recent studies have begun to untangle the intricate mechanisms driving the immune response during normal or atypical, delayed healing. Various *in vivo* models of fracture healing, including genetic knockouts, as well as *in vitro* models of the fracture callus, have been implemented to enable experimental manipulation of the heterogeneous cellular environment. The goals of this review are to (1): summarize our current understanding of immune cell involvement in fracture healing (2); describe state-of-the art approaches to study inflammatory cells in fracture healing, including computational and *in vitro* models; and (3) identify gaps in our knowledge concerning immune-stromal crosstalk during bone healing.

## Introduction

Unlike most tissues in the body, bone has the unique ability to regenerate - this process is dependent on carefully orchestrated crosstalk between immune and stromal cells. Although the term ‘osteoimmunology’ was coined over twenty years ago to describe the role of immune cells in normal and pathological bone remodeling, there is much that remains unknown about mechanisms guiding immune-stromal cell interactions during the process of bone repair (1). With 600,000 yearly cases of malunion or non-union fractures in the US, there is a critical need to understand both restorative and detrimental properties of immune-stromal crosstalk during the fracture healing response (2).

### Overview of fracture healing

**Figure 1:**
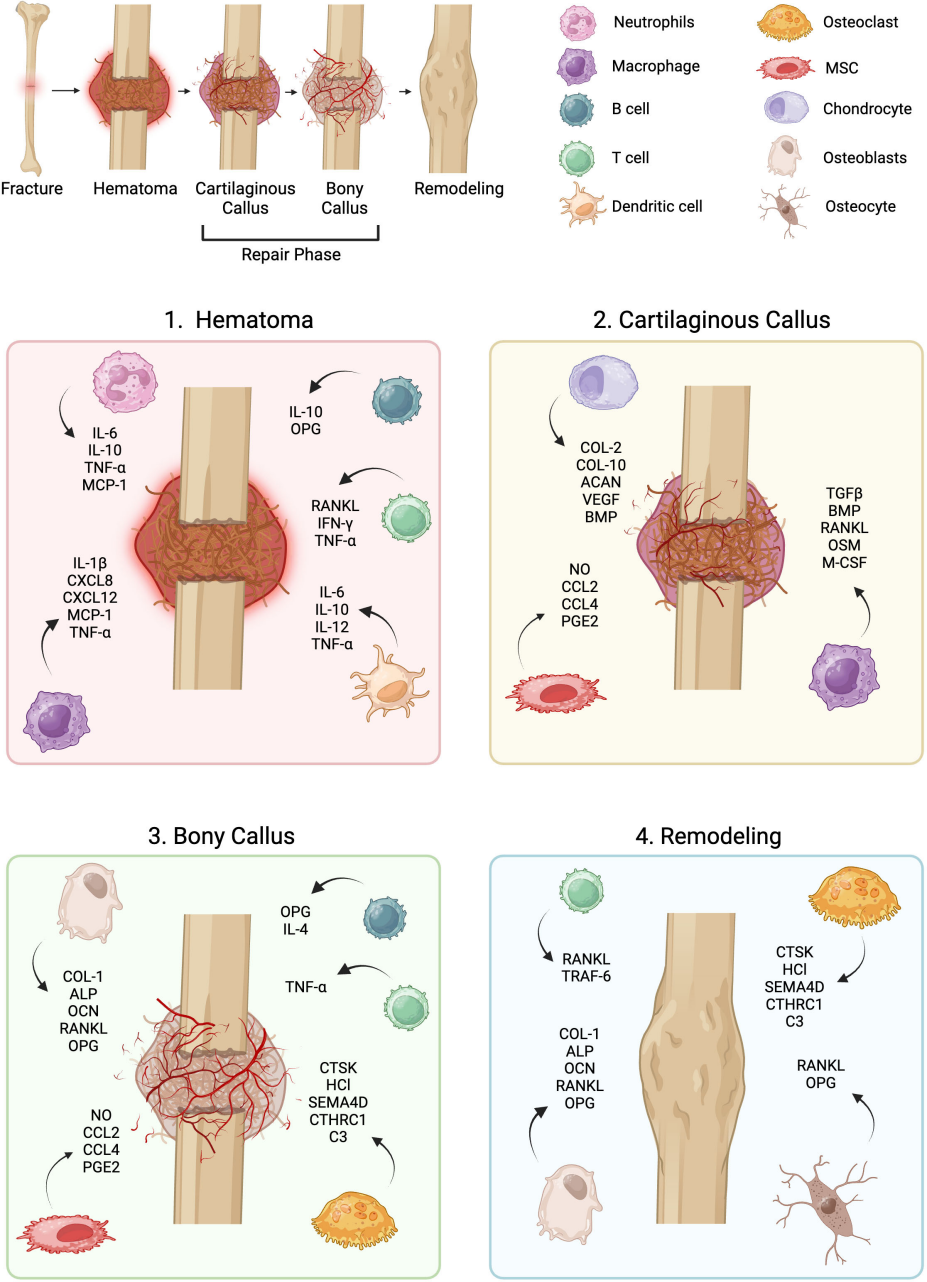
Overview of fracture repair. Fracture repair occurs across distinct phases, each of which involves dynamic stromal-immune cell interactions: the hematoma phase, repair phase, and remodeling phase. MSC, mesenchymal stromal cell.

Fracture repair involves recruitment of immune cells in a temporal and spatial manner that influences the proliferation and differentiation of stromal cells. During the initial stages of long bone callus formation, a fracture hematoma forms, followed by inflammation and stromal progenitor cell recruitment as illustrated in **Figure 1**. Bone formation occurs next via direct, osteoblast-mediated mechanisms (intramembranous ossification) and via indirect, chondrocyte-mediated mechanisms (endochondral ossification) (3). The majority of pre-clinical fracture studies occur in rodents due to feasibility, reproducibility, and similarities in dynamics of fracture healing to that of humans (4).

## Hematoma formation and inflammatory phase

This phase occurs over the first 1-5 days post fracture in humans (5, 6).

### Neutrophils

In the first 24 hours of fracture healing, a hematoma forms and is infiltrated by granulocytic cells (predominantly neutrophils) that act as ‘first responders’ (7, 8). These cells recruit monocytes via secretion of cytokines like interleukins (IL-) 1, 6, and 10; tumor necrosis factor alpha (TNF-α); and monocyte chemoattractant protein 1 (MCP-1) (9–14). Neutrophils have also been implicated in contributing to the initial fibrin-rich clot. Within 48 hours of fracture, neutrophils make up the vast majority of cells present at the injury site and synthesize a fibronectin-containing extracellular matrix (ECM) (15). Fibronectin binds fibrin and provides binding sites for other ECM proteins, cells, and growth factors (16). Neutrophil depletion by anti-Ly6G antibody treatment impairs fracture healing, highlighting the essential role of neutrophils in the early inflammatory response (17). While neutrophil infiltration is key to the formation of the hematoma, sustained neutrophil activation leads to diminished osteogenic activity, reduced callus mineralization, and impaired/delayed healing (18, 19).

### Monocytes/macrophages/dendritic cells

Upon recruitment, systemically-derived monocytes differentiate into macrophages and dendritic cells. Dendritic cells are present during the early phases of fracture healing, and express inflammatory cytokines (IL-6, IL-12, TNF-a, IL-10) (20–24). Furthermore, CD8+ dendritic cells are known to stimulate CD8+ T cells (20). Early on, macrophages remove cellular debris and secrete inflammatory cytokines including IL-1, TNF-α, IL-6, chemokine (C-X-C motif) ligand (CXCL) 8, CXCL12, and MCP-1 (25–27). Macrophage polarization occurs along a spectrum but is often simplified into 3 subclasses: a naïve, pro-inflammatory, or pro-regenerative phenotype. While macrophages are present throughout the healing process, macrophage depletion studies have identified that their presence is most critical in the immediate aftermath of injury during the pro-inflammatory phase (28–31). Polarized macrophages have been shown to exhibit plasticity in their ability to revert back to a naïve resting state *in vitro* (32) and through predictive modeling (33). Inflammatory macrophages demonstrate reduced inducible nitric oxide synthase (iNOS) signaling as time progresses after pro-inflammatory stimulation, eventually returning to a naïve state. While this observation may hold for inflammatory macrophages in tissue repair, it has yet to be described in the context of fracture healing. Macrophage-derived cytokines IL-1β and TNF-α also stimulate fibroblast proliferation within the fracture callus (34). Some studies posit that cytokines, such as TNF-α, secreted by pro-inflammatory macrophages, induce bone morphogenetic protein (BMP) 2, the transcription factor RUNX2, and expression of alkaline phosphatase in mesenchymal stromal cells (MSC) (35, 36). However, other studies suggest that later pro-regenerative macrophages secrete BMP2 and oncostatin M (OSM) to promote ECM mineralization, underscoring the importance of temporal dynamics in fracture healing (37, 38). It has also been demonstrated that during this initial phase pro-inflammatory macrophages secrete vascular endothelial growth factor (VEGF) to stimulate neovascularization. As the pro-inflammatory to pro-regenerative shift occurs, pro-regenerative macrophages secrete platelet-derived growth factor (PDGF) (39). Importantly, although the acute inflammatory phase following hematoma formation is critical for fracture healing, chronic inflammation and persistence of pro-inflammatory macrophages impairs fracture healing (40, 41).

### Natural killer cells

Little is known about the function of natural killer (NK) cells during fracture repair; however, it is hypothesized that they likely assist in debridement of the fracture callus and recruit macrophages to the injury site (9). Early work suggested that NK cell activity was suppressed in fracture patients; whereas recent studies indicate an important role for NK cells in MSC recruitment to the fracture site through neutrophil activating peptide 2 secretion, and in regulation of osteoclastogenesis (42–44). Different classes of NK cells regulate progenitor cell survival during digit tip regeneration that may be comparable to events during fracture healing (45). NK cells also show interdependency with MSC, where MSC secretion of IL-10, transforming growth factor beta (TGF-β), and prostaglandin E2 (PGE2), has been linked to suppression of NK cells (46–48).

### Lymphocytes

Lymphocytes arrive as the initial inflammatory phase wanes. T cells express the pro-osteoclastogenic cytokine, receptor activator of nuclear factor κB ligand (RANKL), whereas B cells express osteoprotegerin (OPG), which blocks RANKL activity, inhibiting osteoclastogenesis (49). Spatio-temporal studies of T and B cells in fracture healing have established increased T cells in the bone marrow immediately after injury, with a significant increase in CD4+ T cells compared to CD8+ T cells. Following this initial spike in T and B cells, they retreat from the injury site, reappearing later during bone formation and remodeling (49). Notably, Reinke et al. determined that CD8+ T cells release interferon ɣ (IFN-ɣ) and TNF-α, and that their persistence throughout the fracture repair process greatly impairs osteoblast differentiation and healing (50). To prevent this, IgM+ CD27+ regulatory B cells release IL-10, suppressing IFN-ɣ, TNF-α, and IL-2 signals from CD8+ T cells to promote resolution of the inflammatory response (51).

## Repair phase

The repair phase occurs between 5 and 21 days in humans and consists of the formation of a cartilaginous soft callus that then converts to a hard bony callus (5). During the repair phase, bone will heal by endochondral ossification, where it goes through a cartilaginous intermediate, or direct intramembranous ossification where MSC differentiate into osteoblasts and deposit a mineralized ECM (31, 52). Both processes are necessary for fracture repair, however the amount that each contributes to healing depends on fracture stabilization and mechanical forces (53). During the soft and hard callus phases, MSC, chondrocytes, osteoblasts, macrophages, osteoclasts, T cells, and B cells are the dominant cell populations (5, 9).

### Macrophages/osteoclasts

Bone-resident macrophages regulate bone formation and play a key role in MSC differentiation. Activated macrophages release the cytokines TGFβ, BMP, and OSM to induce MSC differentiation (54). Chang et al. coined the term ‘osteomacs’ to define a discrete F4/80^pos^ Mac-2^neg/lo^ TRACP^neg^ macrophage population found on the periosteum and endosteum lining the bone (55, 56). Osteomacs promote intramembranous ossification and have been shown to exert control over osteoblast maintenance. Within calvarial cultures, the removal of osteomacs results in decreased mineralization, reduced osteocalcin (OCN) induction, and a limited TNF-α response to LPS, demonstrating an integral role in bone homeostasis and osteoblast function (55, 57). Studies have further demonstrated the importance of the osteomac population in a murine tibia fracture model, where depletion resulted in decreased bone formation (56). During the latter part of the repair phase, inflammatory macrophages, described as F4/80^pos^ Mac-2^pos^ TRACP^neg^ differentiate into osteoclasts through macrophage colony-stimulating factor (M-CSF) and RANKL signaling (56). Osteoclasts can induce osteoblast differentiation through secretion of soluble factors like including collagen triple helix repeat-containing protein 1 (CTHRC1) and complement component C (C3) (58, 59). In contrast to osteomac depletion, depletion of osteoclasts, which resorb cartilaginous ECM through catabolic activity, did not impair bone formation (56). Notably an MSC-derived population of septoclasts have also been recently implicated in cartilage resorption during fracture healing as well as developmental ossification, potentially augmenting this activity. However, septoclast importance in bone remodeling post-fracture is still under investigation (60).

### Lymphocytes

During fracture repair, T and B cells infiltrate the fracture site and assist in osteoblast maturation and retention. In this ‘second-wave’ lymphocytes are absent from the cartilaginous regions of the fracture callus, however they are present near the regions of woven bone (49). Konnecke et al. reported that B cells maintain bone homeostasis through the production of OPG to reduce osteoclastogenesis, and physically interact with osteoblasts to influence their differentiation and function (49). Numerous studies have likewise described T cells as critical for fracture repair (61–65). T cells secrete TNF-α to induce osteogenesis and are necessary for normal deposition of collagen I by osteoblasts during fracture healing (61). T cell depletion further exhibited similar premature mineral deposition as seen in Rag1-deficient mice (which lack mature lymphocytes), pointing toward a T cell-osteoblast interaction pathway (61).

### MSC/chondrocytes/osteoblasts

MSC derive from various sources including the periosteum and bone marrow (66). In the healing callus they begin to differentiate into chondrocytes and osteoblasts. MSC modulate the immune environment by secreting regulatory molecules including nitric oxide (NO) (67), chemokine ligand (CCL) 2 and 4, and PGE2, to recruit macrophages which trigger MSC chondrogenic and osteogenic differentiation (54, 68, 69). Current literature suggests that skeletal MSC derive from multiple sources including the periosteum, endosteum, bone marrow, and vasculature (66). Periosteal-derived MSC at the callus edges have increased osteoblastogenic potential and undergo intramembranous ossification, secreting collagen 1 (COL-1), OCN and alkaline phosphatase (ALP) (70). On the other hand, bone marrow-derived MSC at the fracture site are more predisposed toward endochondral ossification, depositing collagens 2 (COL-2) and 10 (COL-10) as well as sulfated glycosaminoglycans such as aggrecan (ACAN) (10, 71, 72). Under injury conditions, periosteal-derived MSC have also been shown to contribute to endochondral ossification (71). During this process, the cartilaginous callus begins to stimulate vascular infiltration as hypertrophic chondrocytes secrete angiogenic factors VEGF (73), PDGF (74), and placental growth factor (PGF) (75). Vascular infiltration has been demonstrated to be crucial for the replacement of the cartilaginous callus by bone (76). Although immune-derived cues may direct MSC differentiation pathways, recognized contributors to this spatial phenomenon of MSC becoming either osteoblasts or chondrocytes are mechanical cues and hypoxia (40, 77, 78).

## Remodeling phase

This phase typically takes around 18 weeks but can last for up to 1 year under typical fracture healing conditions in humans (5, 79). During fracture remodeling, the initial fracture callus is replaced with mature mineralized tissue and normal bone structure is restored. This coordinated response to injury is the last stage of fracture repair and is the longest, and the least well-studied (80). During the remodeling phase, inflammatory cells (other than osteoclasts) are dramatically reduced, and remodeling is driven by continuous local and systemic cell signaling (81). Bone remodeling occurs as a function of the stresses that bone receives due to forces acting upon it, including muscle actions (82, 83). The ability of bone to remodel post-fracture declines with age in humans. Indeed, children are more likely than adults to experience overgrowth of mineralized tissue, resulting in ectopic bone formation (84). Studies in mice have corroborated the age-related decline in fracture healing potential in humans, showing significant delays in bone remodeling and decreased bone recovery in elderly mice post-fracture (85, 86).

### Osteoclasts

Although osteoclast activity is present early on in fracture repair, it is most prominent in the remodeling phase (87). Osteoclasts work in a balance with osteoblasts and osteocytes to first degrade immature woven bone which is then replaced with more mature bone. Osteoclasts create a reversal zone where the bone surface is eroded, leaving a canopy where osteoprogenitors are found. The basic multicellular unit -an assembly of osteoblasts, osteoclasts, and capillaries- is a prominent hallmark of bone remodeling (81, 88). Osteoclast differentiation is positively regulated by RANKL signaling and negatively regulated by OPG (89). Osteoclasts dissolve bone through secretion of cathepsin K (CTSK) and hydrochloric acid, and degrade ECM via secreted matrix metalloproteinases (90, 91).

### Osteoprogenitors/osteoblasts

MSC differentiate into osteoblasts, which deposit mineral in equilibrium with osteoclast activity (21). Osteoprogenitors and osteoblasts constitute the canopy around blood vessels, serving as the main source of cells contributing to bone formation. A bone remodeling compartment forms near capillaries and sinusoids, providing access to osteoprogenitors including bone lining cells and pericytes (88). Pericytes encircle capillaries, however evidence suggests that these pericytes can migrate to the bone surface and differentiate into mature osteoblasts (92, 93). Osteoblasts secrete RANKL and OPG to modulate osteoclastogenesis (94).

### Lymphocytes

T cells regulate osteoblast-osteoclast equilibrium by secretion of RANKL (95). Although T cell expression of RANKL may drive osteoclastogenesis during bone remodeling, T cells also drive degradation of TNF receptor associated factor 6 (TRAF6), acting as a negative feedback mechanism for osteoclast activity (96).

### Osteocytes

Osteocytes make up 90% of healthy adult bone and function in response to changes in their microenvironment, such as mechanical deformation, to initiate remodeling responses via RANKL and OPG production (97).

## Fracture modeling approaches

The mechanisms by which immune and stromal cells orchestrate fracture repair are not fully understood. To interrogate these complex biological interactions, various models of fracture healing have been developed. Herein follows an overview of models of *in vivo* fracture healing*, in vitro* fracture models, and computational models, to replicate both typical and impaired fracture healing.

### *In vivo* murine fracture model

Animal models most faithfully recapitulate the physiological environment and allow for manipulation of cell responses through genetic knockouts and pharmacological or environmental intervention. Selective ablation of immune cell types in mice has contributed heavily to our understanding of the immune system in fracture healing. Fracture models of comorbidities illustrating immune disruption in fracture healing has been thoroughly reviewed (98–100). Numerous studies have utilized transgenic cre drivers such as LysM-Cre, Mrp8-Cre, and Lck-Cre, as well as Macrophage-Fas Induced Apoptosis (MAFIA) mice to generate immune cell-type specific targeting (101–111). Closed long bone fractures in rodent models are often employed to study fracture healing (112). Factors such as age, ischemia, osteoporosis, and immune deficiency are then incorporated to examine causes of impaired healing (57, 98, 113–117). Fracture in aged populations exhibit increased pro-inflammatory macrophage recruitment as well as increased apoptotic markers in human (118) and mouse (119) systems. Lopez et al. demonstrated that anti-inflammatory modulation of the aged fracture rescues callus formation and healing in aged mice (119). The ischemic fracture model exhibits distinctly smaller callus formation and increased fibrosis (114). Ovariectomy produces postmenopausal osteoporosis in mice, leading to chronic inflammation and increased catabolic activity within bone. Fracture following ovariectomy demonstrates delayed callus mineralization, and remodeling (120, 121). Macrophage populations also exhibit increased IFN-ɣ, nitric oxide, and IL-6 expression (57, 122). Interestingly, MSC isolated from osteoporotic patients do not have impaired potential to regenerate bone, emphasizing the critical role of the immune environment in vivo (123). Multiple studies have revealed that fracture healing is greatly impaired in immunodeficient mice, underscoring the necessity of the immune response in fracture repair (101, 124). While the importance of the innate immune system is indisputable, studies have contested the importance of the adaptive response; Toben et al. demonstrated that eradication of the adaptive immune response using *RAG1^-/-^* mice accelerated fracture healing and improved bone quality (125). However others have stressed the immunoregulatory importance of adaptive immune cells (particularly T cells) in guiding the repair response and enabling osteoblast activity (63, 126). This emphasizes the complexity of the immune response in fracture repair and the necessity for diverse models to better dissect these pathways.

### *In vitro* fracture callus

While the gold standard of preclinical studies is animal models, these models may have limited transferability due to differences in timeline, physiologic structure, pharmacologic response, and variation in specific gene pathways across species, supporting the need for *in vitro* models using human cells and tissues to complement animal work (4, 127, 128). *In vitro* models have been developed over the past decade to create a more physiologically-relevant system for studying human fracture. Along with reducing the number of animals necessary to carry out fracture research, the use of human cells carries additional translational transferability. Pfeiffenberger et al. extensively developed a human-based fracture gap model to interrogate immune-stromal crosstalk *in vitro* (128, 129). While other models, in particular co-culture models (130), focus on later stages of regeneration, this approach uses coagulation of human peripheral blood and MSC to model hematoma development and its progression through fracture repair (129). The hematoma is combined with scaffold-free bone-like constructs made from mesenchymal condensation and allows for manipulation of molecular and environmental cues such as oxygen availability. Hoff et al. developed a human hematoma model using tissue from total hip arthroplasties to monitor and characterize the immune response under bioenergetically-controlled conditions. Cells were exposed to hypoxia with limited nutrients, generating an inflammatory response representative of that seen in fracture after the first 24 hours (131). Increased vascular endothelial growth factor and IL-8 secretion under hypoxia in this model resulted in a decreased granulocytes and increased lymphocytes, as seen *in vivo* (131). Sridharan et al. investigated the interaction of MSC and macrophages in different collagen scaffolds functionalized with hydroxyapatite particles of varying shapes and sizes (132). This emphasized the ability of microenvironmental stimuli to modulate the immune system and presents a unique opportunity to study these interactions in a cell-specific manner. The hydroxyapatite scaffold polarized macrophages toward a pro- or anti-inflammatory phenotype depending upon changes in scaffold particle size and shape, and the authors also demonstrated that macrophage presence increased osteogenesis. Importantly, these studies demonstrate comparable results from an *in vitro* human hematoma model with that shown *in vivo*. *In vitro* models present a powerful tool to understand discrete mechanisms of fracture healing selective to specific cell populations.

### *In silico* fracture modeling

Only recently has computational modeling of fracture healing incorporated intrinsic and extrinsic effects of the immune system, to ascertain their influence on mechanical and biological properties of the callus (133, 134). Computational models are a powerful complementary tool for guiding hypotheses when integrated with *in vivo* and *in vitro* experiments. State-of-the-art *in silico* models encompass continuous, discrete, or hybrid models to interrogate the complex spatiotemporal aspects of fracture healing. No model can holistically capture these processes; however, a corpus of literature is available that aims to help researchers build their own *in silico* models to study the spatiotemporal effects of the immune response on fracture repair (133). Continuous models function at the tissue and cellular level; these models use partial differential equations to create a continuous overview of a given scenario to study inflammation, bone mechanics, and bone repair. Discrete models study specific individual behaviors at the subcellular level, using agent-based approaches or cellular automata models to understand mechanistic processes in response to their environments (133).

Hybrid models aim to bridge the gap from subcellular mechanisms to the tissue. According to Lafuente-Gracia et al., to address the physiologic processes of the inflammatory response, a compartment model is required, where each compartment is assigned its own equation and set of agents (molecules or cells) and transitions (biological processes like phagocytosis or differentiation) between compartments (133).

Kojouharov et al. developed a mathematical model of the early inflammatory response in fracture healing using nonlinear ordinary differential equations (135). It was then elaborated on further in subsequent papers to consider unactivated (M0), classically activated (M1) and alternatively activated (M2) macrophages as separate variables (136) as well as migration due to molecular factors (137). This study was one of the first to incorporate both the primary hematoma formation and the inflammatory response, by identifying the primary entities involved in the early fracture - bone debris, pro- and anti-inflammatory cytokines, macrophages, MSC and osteoblasts. Informing the computational model with the known progression from fracture hematoma to cartilaginous fracture callus to repair, the authors developed a model for differentiation and cytokine production that includes known events such as initial MSC density, debridement rate, proliferation rate, and synthesis of cartilage and bone (135, 136). The model also maintains assumptions such as the inability of M1 and M2 to dedifferentiate back to M0 (136). This model provides an instrument for studying normal and impaired fracture repair, and for extrapolating mechanistic pathways that may otherwise be overlooked and could be adapted clinically to infer the effects of pharmacologics on fracture repair. This group has most recently extended their model to study the direct effects of phagocytes and inflammatory cytokines on macrophage and MSC cell migration during the initial inflammatory and repair phases (137).

Ghiasi et al. also developed a computational model of human fracture with a specific emphasis on the initial inflammatory stage of fracture healing, however they approached it from a mechanobiology perspective (138). This model employs a finite element-based approach that simulates the processes of fracture healing, and the entities present, such as MSC and debris. Both the Kojouharov and Ghiasi models incorporate initial fracture size and cellular density; however, Kojouharov et al. placed emphasis on the cytokines released, while Ghiasi et al. emphasized the Young’s modulus of the granulation tissue along with stresses and mechanical responses that shape hematoma formation and influence callus formation (135, 136, 138).

Most recently, Borgiani et al. developed the COMMBINI model, an agent-based computational model to understand macrophage dynamics that occur during the early inflammatory phase (up to 5 days post-injury) (139). This model utilizes deep-learning algorithms on immunofluorescent stained slides to generate spatial information about different macrophage populations. It uniquely addresses phenotype-specific cell activities (eg. cell proliferation, migration, phagocytosis, apoptosis) and incorporates polarization and cytokine signaling. While the COMMBINI includes neutrophils, the focus of the model is on macrophages, subdivided into categories M0, M1, and M2. To understand the inflammatory phase of fracture repair in guiding healing, the model focuses on expression of key pro-inflammatory and pro-regenerative cytokines like TNFα, IL10, TGFβ, and IFNγ (139). While valuable and an important primary step, the field recognizes that macrophages exist on a spectrum of functionality, more nuanced than discrete M0, M1, or M2 states.

## Discussion

Fracture repair is a complex process orchestrated by immune and stromal cells to regenerate bone tissue. Inducing ischemia results in aberrant repair and regeneration of tissues, underscoring the importance of systemic immune cells to guide healing (140, 141). Studies involving the effect of limb ischemia on fracture healing date back to the 1960s and while the importance of the immune response during fracture repair is well acknowledged, immune alterations in fracture healing under ischemic conditions remain unclear (142). This is true for other impaired healing conditions as well, for instance in aged models or diabetic models where there is increased systemic inflammation. Numerous methods for inducing and modulating fracture repair have been developed to study tissue healing and remodeling *in vivo* – including the integral roles of immune cells. *In vitro* systems allow for the study of mechanisms in discrete phases and specific cell interactions, with the advantage of utilizing human cells. Computational models enhance our study of fracture healing by expanding upon our understanding of networks underlying the fracture microenvironment and simulating the healing response. Importantly, they serve as a tool to study pharmacologic intervention in fracture repair, in conjunction with *in vivo* and *in vitro* models. Used together, these models provide a powerful and holistic approach for interrogating immune dynamics and mechanisms in normal and impaired fracture healing, and will continue to evolve and incorporate more complex variables.

## Author contributions

CC: Conceptualization, Writing – original draft, Writing – review & editing. KH: Conceptualization, Writing – review & editing. AK: Conceptualization, Writing – review & editing.
